# Assessing perceptions of stigmatization by others for seeking help in China: psychometric characteristics and measurement invariance across gender and therapy experience

**DOI:** 10.3389/fpsyg.2024.1391239

**Published:** 2024-05-30

**Authors:** Kun Cheng, Wenxian Yang, Yan-Hong Zhang, Zhuang She

**Affiliations:** ^1^School of Foreign Studies, Research Center for Social Psychology of Yangtze University, Jingzhou, Hubei, China; ^2^Center of Mental Health Education and Counseling, Lanzhou University, Lanzhou, Gansu, China; ^3^College of Education and Sports Sciences, Research Center for Social Psychology of Yangtze University, Jingzhou, Hubei, China; ^4^Department of Psychology, School of Social and Behavioral Sciences, Nanjing University, Nanjing, China

**Keywords:** stigma, help-seeking, social network stigma, reliability, validity, invariance

## Abstract

The stigma of social networks may be more noticeable in collectivist societies like China, but research in this area has largely been overlooked due to a lack of reliable measurement. To address this gap, this study tested the psychometric properties of the Chinese version of the Perceptions of Stigmatization by Others for Seeking Help (PSOSH) scale in the Chinese general population, and tested its invariance across gender and prior therapy experience. In a national online survey, 640 adults completed the PSOSH and conceptually related scales: Self-stigma of Seeking Help (SSOSH), Stigma of Seeking Professional Psychological Help Scale (SSPPH, i.e., public stigma) and Attitudes toward Seeking Professional Psychological Help Scale-Short Form (ATSPPH-SF). Confirmatory factor analysis supported the unidimensional structure of the original PSOSH. The Cronbach’s α coefficient was 0.84 and the 3-week test–retest reliability of was 0.77. The PSOSH showed moderate correlations with the three conceptually related scales, supporting its concurrent validity. Importantly, the PSOSH significantly predicted self-stigma scores, even when considering demographic variables and public stigma, supporting its incremental validity. The scale also demonstrated scalar invariance across gender and across subgroups who had vs. did not have previous therapy experience, supporting comparisons of latent means across these groups. The PSOSH is a reliable and valid instrument for assessing social network stigmatization of professional help-seeking in Chinese community samples.

## Introduction

Stigma is a crucial barrier to psychological help-seeking (Corrigan, 2004; Vogel et al., 2013a). Two types of stigma have been extensively studied: public stigma and self-stigma (for a review, see Corrigan, 2004). Public stigma refers to the external form of stigma, which reflects the general social group’s negative stereotype of a specific stigmatized group (Corrigan, 2004). For instance, the social group might hold the stereotype that a person seeking professional psychological counseling services are unwelcome or socially unacceptable. In contrast, self-stigma is an internal form of stigma that involves low self-evaluation and self-inefficiency due to external public stigma (Vogel et al., 2006). For example, people may believe that if they seek counseling services they are socially unacceptable (Vogel et al., 2007).

Individuals who perceive greater public or self-stigma about the professional help-seeking have been shown to have lower awareness of mental health problems, a more negative attitude toward professional psychological counseling services, and a lower willingness or intention to seek counseling (Vogel et al., 2007; Bathje and Pryor, 2011; Lannin et al., 2015). The Internalized Stigma Model (Lannin et al., 2015) suggests that public stigma and self-stigma influence professional help-seeking through different paths. Whereas self-stigma has a direct negative effect on help-seeking, public stigma influences the individual’s intention to seek help through the mediating effect of self-stigma (Vogel et al., 2007; Brenner et al., 2020). However, less is known about the effects of stigma from the person’s immediate circle (e.g., friends and family; Vogel et al., 2009). Vogel et al. (2009) introduced the concept of social network stigma, which they defined as the stereotypes about professional help-seeking held by groups in direct contact with the individual (Vogel et al., 2009). If the person perceives negative reactions from members of their personal network, then this stigma might have a cumulative effect on public stigma, further reducing the probability of their seeking psychological services (Vogel et al., 2009).

Social network stigma may be more prevalent in collectivist cultures than in other cultures. Collectivist cultures place high importance on social connections and family relationships, leading individuals to be heavily influenced by family and friends when seeking psychological help (Topkaya et al., 2017). Thus, social network stigma may be a strong deterrent against psychological help-seeking in the collectivist culture of China. Consequently, examining social network stigma in China could be beneficial in developing interventions that promote psychological help-seeking in this cultural context.

Vogel et al. (2009) created the English language Perceptions of Stigmatization by Others for Seeking Help (PSOSH) scale to evaluate the impact of social network stigma on psychological help-seeking in a western sample. The scale was designed to measure the degree of stigma individuals experience from their social network, such as friends and family. The PSOSH has shown sound psychometric characteristics, with an internal consistency coefficient of 0.91 and three-week retest reliability of 0.82 (Vogel et al., 2009). Confirmatory factor analysis showed that the PSOSH has a one-dimensional structure and is moderately correlated with total scores on the construct of public stigma (Vogel et al., 2009; Topkaya et al., 2017). Moreover, the PSOSH could significantly predict self-stigma even when controlling for public stigma (Topkaya et al., 2017). These findings suggest that social network stigma and public stigma are distinct constructs. Consequently, social network stigma has been tested in several studies as a negative predictor of psychological help-seeking attitudes and intentions (Ludwikowski et al., 2009; Choi and Miller, 2014; Bird et al., 2020). Given that social network stigma is essentially an external form of stigma, it is consistent with the Internalized Stigma Model (Lannin et al., 2015) that its impact on psychological help-seeking is firstly through an increase in an individual’s self-stigma, which in turn affects psychological help-seeking outcomes (Choi and Miller, 2014).

One understudied issue regarding the English-language PSOSH is its measurement invariance. Currently, only one study has investigated the invariance of the English-language version of the PSOSH across cultures (Vogel et al., 2019). The researchers found metric invariance across 11 European, American and East Asian countries/regions, but not scalar invariance, which is an essential condition for valid comparisons of means across groups (Putnick and Bornstein, 2016; Vogel et al., 2019). Given the small number of studies in invariance analysis of the PSOSH (Vogel et al., 2019), coupled with non-optimal findings in the previous test of scalar invariance, we tested if the PSOSH was scalar invariant across gender and across previous therapy experience (with vs. without therapy experience). Because gender and therapy experience have been shown to be associated with the stigma of professional help-seeking (Tang and Wen, 2015), it is necessary to establish the measurement invariance between these subgroups before making meaningful between-group comparisons.

A potential reason for the lack of research on the effect of social network stigma on psychological help-seeking in China is that the psychometric properties of the Chinese version of the PSOSH (PSOSH-C) have not been fully tested. There were four objectives in this research on the Chinese version of the PSOSH: (a) to test the internal reliability and test–retest reliability; (2) to investigate its factor structure, convergent validity (with other related measures), and incremental validity (controlling for public stigma); (3) to examine its measurement invariance across participants’ gender (men and women) and previous therapy experience (had and had not been in therapy).

## Methods

### Participants and procedure

We employed a commercial research firm (powered by Wenjuanxing, a platform providing functions equivalent to Survey Monkey) to construct a national sample and collect data online. Wenjuanxing possesses a vast database containing data from more than 2.6 million registered Chinese users nationwide. With customers’ consent, Wenjuanxing selected potential participants to form a nationally representative sample of adult community, considering gender, age, education, and region. These potential participants were then contacted via email and provided with information about the study.

On the first page of the survey, participants were informed of the research aim, the completion time (about 8 min), and the anonymity of their responses to the questionnaires. Participants gave informed consent and were then directed to the formal survey interface. The participants received about USD 0.8 for completing the survey, and those who returned three weeks later to provide test–retest reliability data received an additional about USD 0.15. This study was approved by the Research Ethics Committee of the first author’s institution.

A total of 643 individuals took part in the study during the seven-day period of data collection. However, three participants under 18 were excluded from the final analysis. The final sample included 640 participants, of whom 344 were men and 296 were women. Participants’ ages ranged from 18 to 73 years, with a mean age of 30.7 (*SD* = 8.2). About 94% of the participants had completed college or higher, 81.1% were currently employed, 64.8% were married, and 54.8% had prior therapy experience. To assess test–retest reliability, 46 participants (28 men; average age = 30.1 years) took the PSOSH a second time after a three-week interval.

### Measures

#### Perceptions of stigmatization by others for seeking help scale

The 5-item PSOSH assesses people’s perception that people with whom they have close relationships have negative attitudes about seeking help from a mental health professional (Vogel et al., 2009). The questionnaire instructs participants to “Imagine you had a problem that needed to be treated by a mental health professional. If you sought mental health services, to what degree do you believe that the people you interact with would _____?” The representative items include “Think of you in a less favorable way.” Each item is rated on a Likert-type scale from 1 (not at all) to 5 (a great deal).

The total score is calculated by adding all five items, with a higher score indicating greater perceived stigma from those with whom the person interacts. The Chinese version of the PSOSH, provided by the original author, which was used in one previous comprehensive research (Vogel et al., 2019). However, the reliability and validity of the Chinese version of the PSOSH have not been fully tested. We used the Chinese version as Vogel et al. (2019) used in the present study and tested its psychometric properties in the general population in China.

#### Self-stigma of seeking help scale

The SSOSH scale was designed to assess the potential threats to an individual’s self-evaluation (i.e., self-stigma) when they consider seeking psychological help (Vogel et al., 2006). The scale consists of ten items rated on a 5-point Likert scale from 1 (strongly disagree) to 5 (strongly agree). The representative items include “I would feel inadequate if I went to a therapist for psychological help” and “My self-esteem would increase if I talked to a therapist.” The total score is calculated by reverse-coding the five positive items and adding the scores for all items. Higher scores on this scale indicate a higher level of concern regarding the potential negative impact that seeking help from a mental health professional may have on one’s self-esteem, self-satisfaction, self-confidence, and overall sense of self-worth. The Chinese version of the self-stigma of seeking help scale showed good reliability and validity in a Chinese sample (Vogel et al., 2013b). In this study, the internal consistency (Cronbach’s alpha, same below) of the scale was 0.73.

#### Stigma of seeking professional psychological help scale

The SSPPH scale was designed to measure individuals’ perceptions of the public stigma attached to seeking professional help (Komiya et al., 2000). This scale consisted of 5 items. The representative items include “seeing a psychologist for emotional or interpersonal problems carries social stigma” and “people tend to like less those who are receiving professional psychological help.” Participants were asked to rate each item on a 4-point Likert scale ranging from 1 (strongly disagree) to 4 (strongly agree). The total score is calculated by adding all five items, with higher scores indicating greater perceived public stigma. The Chinese version of the SSPPH scale showed good reliability and validity in a Chinese sample (Zhou et al., 2019). In this study, the internal consistency of the scale was 0.76.

#### Attitudes toward seeking professional psychological help scale-short form

The ATSPPH-SF was developed to evaluate a person’s attitudes toward professional help-seeking (Fischer and Farina, 1995). The scale consists of 10 items. The representative items include “If I believed I was having a mental breakdown, my first inclination would be to get professional attention” and “Considering the time and expense involved in psychotherapy, it would have little value for a person like me.” The participants were asked to rate each item on a 4-point Likert scale ranging from 0 (disagree) to 4 (agree). The total score is calculated by reverse-coding the five negative items and adding the scores for all items. Higher scores indicate more a positive attitude toward professional psychological help-seeking. The Chinese version of the ATSPPH-SF showed good reliability and validity in a Chinese sample (Shu et al., 2019). In this study, the internal consistency of the scale was 0.69.

### Data analysis

The data analyses were conducted using the IMB Statistical Package for the Social Sciences software 21.0 (SPSS 21.0) and Mplus 8.3.

Descriptive statistics, internal consistency reliability, correlations, and regression analyses were calculated in SPSS 21.0. Internal consistency reliability was evaluated by Cronbach’s α coefficient; the concurrent validity was assessed by calculating the Pearson’s coefficient between the total score of the PSOSH scale and scores on three measures of other stigma-related variables (i.e., self-stigma, public stigma, and attitudes toward help seeking).

Confirmatory Factor Analysis (CFA) was used to test whether the Chinese version of the PSOSH scale showed the same unidimensional construct as the original English version proposed by Vogel et al. (2009). We used the following indicators of model fit: Comparative Fit Index (CFI), Tucker–Lewis Index (TLI), root mean square error of approximation (RMSEA), and standardized root mean square residual (SRMR). The fit was considered good if RMSEA was below 0.06, SRMR was below 0.08, and CFI and TLI were above 0.95 (Hu and Bentler, 1999). The parameter estimates for the CFA were obtained using the maximum likelihood method (ML), given the normal distribution of the data (see below).

Consistent with prior work (She et al., 2023b), we tested three models with increasing constraints to assess configural, metric, and scalar invariance. In one set of analyses, the results for men were compared to those for women; in the other set, the results for participants who had been in therapy were compared to those who had not. First, to assess configural invariance we conducted factor analysis in each subgroup without imposing any constraints to examine whether the PSOSH scale had the same factor structure across subgroups (Model 1). Second, we tested for metric invariance by determining whether the factor loadings in the factor analyses were equivalent across the subgroups (Model 2). Meeting the criteria for metric invariance indicated that the PSOSH was consistent across different groups, allowing for valid comparisons of factor variance and structural relationships between groups (Chen et al., 2020).

Third, we scrutinized scalar invariance by assessing whether the PSOSH scale displayed the same item intercepts across subgroups (Model 3). Meeting the criteria for scalar invariance implies that PSOSH scores from various groups share a common origin and unit of measurement. Consequently, variations in observed item means among groups can be ascribed to disparities in latent factor means (Chen et al., 2020). Finally, full mean invariance was tested across participants’ gender and therapy experience in order to determine if there were any significant differences in PSOSH scores based on these variables.

Invariance was assessed by measuring the change in fit compared to the previous level of measurement invariance (i.e., metric vs. configural invariance and scalar vs. metric invariance): △CFI and ΔRMSEA values were used to evaluate the model fit with increased constraints. We utilized the recommended cutoff of |ΔCFI| ≤ 0.01 and |ΔRMSEA| ≤ 0.015 to indicate invariance in specific model fit indices (Cheung and Lau, 2012).

## Results

### Descriptive statistics and item analysis

Descriptive statistics for the Chinese version of the PSOSH items can be found in Table 1. Both the skewness and kurtosis values, in absolute terms, were below 2, suggesting a normal distribution. We also computed the correlations between each individual item and the total score of the scale. The findings indicated a strong correlation between each of the five items and the overall scale score, with correlation coefficients (*r*) ranging from 0.77 to 0.81.

**Table 1:** Descriptive statistics for the five-item PSOSH measure.

Items	*M*	*SD*	Skewness	Kurtosis
1. React negatively to you	2.19	0.97	0.92	0.66
2. Think bad things of you	2.27	0.98	0.72	0.28
3. See you as seriously disturbed	2.47	1.15	0.34	−0.81
4. Think of you in a less favorable way	2.17	1.14	0.81	−0.20
5. Think you posed a risk to others	1.83	1.05	1.25	0.86

### Confirmatory factor analysis

The results of the Confirmatory Factor Analysis (CFA) showed that the one-dimensional structure of the 5-item PSOSH-C had an excellent fit to the data: CFI = 0.997, TLI = 0.994, RMSEA = 0.033, and SRMR = 0.012. All items had salient loadings (i.e., > 0.30; Brown, 2015), and the CFA factor loadings are presented in Table 2.

**Table 2:** Standardized factor loadings for the data (*N* = 640).

Cronbach’s α	Item 1	Item 2	Item 3	Item 4	Item 5
0.84	0.799***	0.746***	0.657***	0.693***	0.705***

***p < 0.001.

### Reliability

The PSOSH-C demonstrated strong internal reliability with a Cronbach’s α coefficient of 0.84. Moreover, its test–retest reliability over a three-week interval (*n* = 46) was recorded as 0.77.

### Concurrent validity

We evaluated the concurrent validity of the PSOSH by examining its correlations with three other stigma-related measures: the Public Stigma scale, the Self-Stigma scale, and the Attitudes toward Seeking Professional Psychological Help Scale-Short Form (ATSPPH-SF). The PSOSH total scores showed a moderate positive correlation with the Public Stigma scale and the Self-Stigma scale. See more details in Table 3.

**Table 3:** Correlations between PSOSH and related variables.

	Self-stigma	Public stigma	Attitude toward help-seeking
PSOSH	0.46***	0.62***	−0.38***

***p < 0.001.

### Incremental validity

A hierarchical regression analysis was performed to assess the incremental validity of the PSOSH scale, with self-stigma as the dependent variable. In Step 1, the control variables of gender, age, educational background, and previous therapy experience were entered. In Step 2, public stigma was added. In Step 3, social network stigma was added. The results showed a significant regression coefficient for social network stigma as a predictor of self-stigma in Step 3, after controlling for public stigma in Step 2 (B = 0.18, *p* < 0.01). Furthermore, the explained variance showed a significant increase from Step 2 to Step 3 (ΔR^2^ = 0.01, *p* < 0.01).

### Measurement invariance

Table 4 shows that full scalar invariance was supported across the two gender subgroups and across the two therapy experience subgroups, using models with augmented constraints (i.e., configural invariance, metric invariance and scalar invariance). No significant deterioration was observed in the comparison of the metric and scalar model between tested subgroups, as substantiated by the acceptable absolute values of ΔCFI (< 0.01) and ΔRMSEA (≤ 0.015) across all tested invariance analyses (see Table 3 for model comparisons). Consequently, the results provide robust support for full scalar invariance of the PSOSH scale across the gender subgroups and across the subgroups defined by previous psychotherapy. These findings indicated that participants in each pair of subgroups had similar responses to the PSOSH items, and they justify subsequent latent mean comparisons across tested groups.

**Table 4:** Measurement invariance across gender and prior therapy experience.

	*χ^2^*	df	CFI	RMSEA	*M* comparison	ΔCFI	ΔRMSEA
*Across gender*							
M1. Configural invariance	23.935	10	0.988	0.066	─	─	─
M2. Full metric invariance	30.661	14	0.986	0.061	M2-M1	−0.002	−0.005
M3. Full scalar invariance	38.781	18	0.983	0.060	M3-M2	−0.003	−0.006
M4. Latent mean invariance	40.115	20	0.983	0.056	M4-M3	0.00	−0.004
*Across therapy experience*							
M5. Configural invariance	19.596	10	0.992	0.055	─	─	─
M6. Full metric invariance	20.951	14	0.994	0.040	M6-M5	0.002	−0.015
M7. Full scalar invariance	27.573	18	0.992	0.041	M7-M6	−0.002	0.001
M8. Latent mean invariance	30.572	20	0.991	0.041	M8-M7	−0.001	0.00

The results of the further test of latent mean invariance suggest that there were no significant differences in PSOSH scores based on participants’ gender and therapy experience. This was evidenced by the lack of significant deterioration in the comparison of the scalar model and latent mean model between the tested subgroups.

## Discussion

This study tested the psychometric properties of the Chinese version of the PSOSH in the general population. The PSOSH showed robust properties, demonstrated by multiple measures of reliability and validity, and the same one-factor structure in the original English language version developed in the United States (Vogel et al., 2009). This consistency underscores that the concept of social network stigma might be global in nature, transcending Western contexts and resonating in non-Western cultures as well.

While the Chinese version of the PSOSH demonstrated good excellent internal consistency, with a Cronbach’s α coefficient surpassing 0.80, it is worth noting that the α coefficient observed in this study was somewhat lower than that reported by Vogel et al. (2009), where it exceeded 0.90. This discrepancy may stem from the sampling techniques employed. The original measure (Vogel et al., 2009) was developed in younger and more homogenous samples of college students in one U.S. city. In contrast, our study drew from a more representative pool of adults from many provinces in China. The 3-week test–retest reliability was similar in our study and in the original study (Vogel et al., 2009). Overall, our research lends support to the reliability of the PSOSH within the general Chinese general population.

The PSOSH total score showed significant moderate correlations with scores on the Self-Stigma Scale, Public Stigma Scale, and Professional Psychological Help-Seeking Attitude Scale. These correlations bolster the concurrent validity of the PSOSH. Notably, the PSOSH scale’s moderate association with the Public Stigma Scale aligns with prior research that distinguished social network stigma from public stigma (Vogel et al., 2009; Topkaya et al., 2017).

Further analyses revealed that even after controlling for variables such as gender, age, education, and previous counseling experience, along with public stigma, social network stigma remained a significant predictor of self-stigma. This suggests that social network stigma may uniquely influence individuals’ attitudes toward seeking psychological help (Vogel et al., 2009; Sezer and Kezer, 2013; Bird et al., 2020). In other words, beyond the broadly perceived public stigma, an individual’s perception of social network stigma acts as a supplementary factor in the internalization of stigma associated with seeking professional mental health services (Sezer and Kezer, 2013).

The multi-group confirmatory factor analyses revealed full invariance both across gender and across groups based on prior psychotherapy experience. This suggests that the mean scores for all PSOSH items were statistically consistent across these subgroups. This allows for meaningful latent mean comparisons between men and women, as well as between individuals with and without prior therapy experience.

We found no significant differences in PSOSH scores across gender or prior therapy experience. This indicates that both genders might perceive a similar level of stigma from their social networks. Surprisingly, the lack of a difference between participants with and without prior therapy experience diverged from our expectations. We had anticipated that individuals with previous therapy exposure would perceive less social network stigma, given their engagement with professional mental health services (Pettigrew and Tropp, 2000). Yet this finding becomes understandable when acknowledging the enduring nature of external stigma (e.g., social network stigma) and the challenges associated with altering it within broader social interactions (Brenner et al., 2020). This understanding makes sense when considering the generally held intense social stigma against professional counseling services in China (Kim and Zane, 2016; Li et al., 2016). Moreover, Chinese clients in therapy have been shown to attend a limited number of sessions, with the mean number of sessions less than 4.7 across adult clients from outpatient and counseling settings in China (She et al., 2023a). Also, they may quit early because of social network stigma. Overall, the short psychotherapy experience may also make it difficult to change their perception of stigma from others. More studies are needed to investigate this issue in the Chinese cultural context.

Our research provided a reliable tool for evaluating the perceived social network stigma among Chinese individuals. In a collectivist culture that places a strong emphasis on social connections and family ties, individuals may place greater importance on the opinions of their close friends and relatives when seeking professional help, as opposed to their Western counterparts (Topkaya et al., 2017). Therefore, interventions aimed at reducing the perceived stigma among Chinese individuals regarding seeking help from those close to them could prove to be an effective strategy for enhancing help-seeking outcomes.

### Limitations

There are several limitations to our study. First, our sample predominantly consisted of young adults with a bachelor’s degree or above. This does not reflect the broader Chinese demographics. Consequently, the generalizability of our findings to populations with less formal education, older adults, and adolescents remains uncertain. More diverse and representative samples (e.g., varied in age and educational levels) are needed to replicate our findings. Second, while this study assessed the multi-group invariance of the PSOSH, it did not delve into longitudinal invariance, which has not been tested in the existing literature. Future research is encouraged to examine this aspect, as it is pivotal to ensuring the validity of mean comparisons in longitudinal tracking studies.

Another limitation is the representativeness of the sample. By using an online survey, we may have excluded individuals who do not have access to technology or are uncomfortable using it. This could introduce bias into our sample. To address this limitation, future studies could consider using a combination of paper questionnaires and online surveys to obtain a more representative sample for research purposes.

## Conclusion

This study furnishes evidence supporting the use of PSOSH in Chinese general population. Considering the distinct cultural nuances associated with seeking professional help, we advocate for further research using the PSOSH to study social network stigma in non-Western contexts, particularly in collectivist societies. Future studies could build on this research on perceptions by examining help-seeking behaviors through the lens of social network stigma.

## Data availability statement

The raw data supporting the conclusions of this article will be made available by the authors, without undue reservation.

## Ethics statement

The studies involving humans were approved by Centre for Mental Health Education of Yangtze University. The studies were conducted in accordance with the local legislation and institutional requirements. The participants provided their written informed consent to participate in this study. Written informed consent was obtained from the individual(s) for the publication of any potentially identifiable images or data included in this article.

## Author contributions

KC: Conceptualization, Investigation, Writing – original draft. WY: Conceptualization, Formal analysis, Writing – review & editing. Y-HZ: Conceptualization, Writing – review & editing, Funding acquisition, Investigation. ZS: Conceptualization, Investigation, Data curation, Formal analysis, Writing – original draft, Writing – review & editing.
